# Triage tool for suspected COVID-19 patients in the emergency room: AIFELL score

**DOI:** 10.1016/j.bjid.2020.07.003

**Published:** 2020-08-20

**Authors:** Ian Levenfus, Enrico Ullmann, Edouard Battegay, Macé M. Schuurmans

**Affiliations:** a University Hospital Zurich, Division of Internal Medicine, Zurich, Switzerland; b University of Zurich, Faculty of Medicine, Zurich, Switzerland; c University of Leipzig Medical Center, Psychotherapy and Psychosomatics, Department of Pediatric Psychiatry, Leipzig, Germany; d Technische Univeristat Dresden, Department of Medicine, Dresden, Germany; e South Ural State University, Department of Medical Biology, Chelyabinsk, Russia; f University Hospital Zurich, Division of Pulmonology, Zurich, Switzerland

**Keywords:** COVID-19, SARS-CoV-2, Score, Emergency

## Abstract

Clinical prediction scores support the assessment of patients in the emergency setting to determine the need for further diagnostic and therapeutic steps. During the current COVID-19 pandemic, physicians in emergency rooms (ER) of many hospitals have a considerably higher patient load and need to decide within a short time frame whom to hospitalize. Based on our clinical experiences in dealing with COVID-19 patients at the University Hospital in Zurich, we created a triage score with the acronym "AIFELL" consisting of clinical, radiological and laboratory findings.

The score was then evaluated in a retrospective analysis of 122 consecutive patients with suspected COVID-19 from March until mid-April 2020. Descriptive statistics, Student's *t*-test, ANOVA and Scheffe's post-hoc analysis confirmed the diagnostic power of the score. The results suggest that the AIFELL score has potential as a triage tool in the ER setting intended to select probable COVID-19 cases for hospitalization in spontaneously presenting or referred patients with acute respiratory symptoms.

Due to the worldwide spread of SARS-CoV-2 and rapidly increasing numbers of infections, the novel coronavirus became a considerable strain for emergency rooms (ER), especially when several suspected cases with unspecific general or respiratory symptoms arrive at the same time. Identification of more critical patients in the ER for hospitalization is a challenge since the detection of SARS-CoV-2 in nasopharyngeal swabs by quantitative polymerase chain reaction (qPCR) still requires many hours (>6 h in our setting). Therefore, the qPCR result currently cannot be used in the frontline setting to decide whom to hospitalize and who can be managed as an outpatient. Rapid point of care tests for SARS-CoV-2 were being developed at the time of the study but were not validated for routine use.^1^

As a frontline physician, whose task was to evaluate and triage patients arriving with symptoms suggesting COVID-19 in the ER coronavirus unit of the University Hospital in Zurich, the first author was confronted with the problem of whom to choose for hospitalization due to probable COVID-19 and whom to discharge whilst the qPCR results of the swab were pending.

The hospitalization criterion was to select patients at risk for developing more severe symptoms leading to respiratory failure (COVID Stages II or III^2^). During clinical routine work in the frontline unit, the question of a score arose to support the triage process and to assist other physicians in similar situations.

We therefore followed up consecutively hospitalized patients with proven COVID-19 in order to determine initial features which may help distinguishing probable COVID-19 cases from other respiratory problems. In 30 personally encountered consecutively hospitalized patients with qPCR-proven COVID-19 studied initially as a pilot cohort, we found that elevated C-reactive protein (CRP) and lactate dehydrogenase (LDH) levels as well as lymphocytopenia were characteristic laboratory patterns. Evidence obtained from literature searches using the keywords “COVID-19”, “SARS-CoV-2”, “laboratory” and “patients” in PubMed proved that these laboratory abnormalities were associated with COVID-19.^3^, ^4^, ^5^ Additionally, most patients in the pilot cohort presented with an elevated body temperature and showed unilateral or bilateral pulmonary infiltrates in conventional chest radiography. Several patients mentioned spontaneously having noted an attenuation of smell or taste and this symptom, although not widely recognized as a typical feature at the time, was considered to be COVID-19-associated.^6^ When the relevant paraclinical components were analyzed, cut-off values became more evident and a simple score was created based on typical clinical information routinely available in our ER.

The AIFELL score includes an **A**ltered sense of smell/taste, **I**nflammation (C-reactive protein ≥30 mg/L), radiological **I**nfiltrates, **F**ever (≥38.0 ° C), **E**levated **L**actate dehydrogenase (LDH) levels (>400 U/L) and **L**ymphocytopenia (absolute count <1.45 G/L). The score is calculated by adding the number of criteria met at initial presentation in the ER, whereas each criterion equals one point (score range from 0 to 6 points).

To assess the score, we applied it retrospectively to consecutive patients with suspected COVID-19 admitted via the ER from March until mid-April 2020. Only those cases evaluated with chest imaging and a blood test including at least two of the three considered blood parameters at presentation in the ER, who did not decline the general research consent, were included. Of 122 patients with suspected COVID-19, 52 cases turned out to have other respiratory problems.

SARS-CoV-2 positive patients (*n* = 70) were classified according to the stages suggested by Siddiqi and Mehra (Supplementary Figure S.1).^2^

The study was approved by the Institutional Review Board of the Canton of Zurich (Cantonal Ethics Committee, Nr. 2020-00854). After testing for normal distribution and standardized outliers, we created a new variable named “paraclinical measurements” by including the z-standardized mean values of LDH, CRP, inversely poled serum lymphocytes and auricular body temperature. We afterwards summarized this variable with lung infiltrates seen by imaging and alterations of smell/taste indicated by patient history to get our predictor score named “AIFELL”. Student's *t*-tests and an ANOVA with Scheffe's post-hoc tests were performed for group comparisons. For all analyses, we used MS Excel 2016 and SPSS 24.

The mean age of our SARS-CoV-2 positive subjects (*n* = 70) was 60.6 years ± 14.2 (standard deviation) vs. 57.9 years ± 17.9 of our SARS-CoV2 negative subjects (*n* = 52; *t* = .908; *p* = 0.37). There were significantly different AIFELL scores before and after *z*-standardization (*t* = 5.77, *p* < 0.001) between SARS-CoV-2 positive patients (mean = 2.43 ± 0.15 standard error) and SARS-CoV-2 negative patients (mean = 1.30 ± 0.12) (Supplementary Figure S.2).

A score of ≥4 points/criteria met at presentation was highly associated with qPCR-based SARS-CoV-2 detection in nasopharyngeal swabs and development of symptomatic COVID-19 (Stages II or III), thus justifying hospitalization. Scores between 0 and 3 were associated with other respiratory conditions (Table 1).

**Table 1:** Distribution of included patients and their clinically assigned AIFELL scores.

**Consecutive patients with suspected COVID-19 admitted via the ER from March through mid-April 2020 (n = 122; 72 males, 50 females)**				
**Nasopharyngeal qPCR result**	**SARS-CoV-2 positive** (n = 70, age 60.6 years ± 14.2)			**SARS-CoV-2 negative** (n = 52, 57.9 years ± 17.9)
**Components of the AIFELL score:**Altered smell or taste (yes/no), Inflammation (CRP ≥ 30 mg/L), Infiltrates (yes/no), Fever (≥ 38° C), Elevated LDH (>400 U/L), Lymphocytopenia (absolute count < 1.45 G/L)				
**Diagnosis**	**COVID-19 Stage I (n = 10)**	**COVID-19 Stage IIa (n = 31)**	**COVID-19 Stage IIb (n = 29)**	Other respiratory problems like exacerbated COPD, bronchial asthma, bacterial pneumonia, aspiration pneumonitis, other viral infections (influenza, metapneumovirus), cardiac failure
**Mean AIFELL score**	1.8 ± 0.8	4.6 ± 0.8		2.2 ± 1.1
	4.19 ± 1.28			
**Total number n AIFELL positive (4–6 points)**	0	29 (93.5%)	29 (100%)	5 (9.6%)
**Total number n AIFELL negative (0 – 3 points)**	10 (100%)	2 (6.5%)	0	47 (90.4%)

*Legend*: Results given as mean ± SD unless indicated otherwise. Stage III patients with progressive systemic inflammation were usually admitted directly to ICU from normal ward or other hospitals in our setting, not through the ER. Therefore, they are not mentioned in this table. SD, standard deviation.

Stage III patients (severe disease) with extra-pulmonary systemic hyperinflammation, ARDS or symptoms of shock were usually transferred to intensive care unit (ICU) from normal wards or from other hospitals. Documented cases of ICU transfers of patients who deteriorated in the course of disease (progression from Stage II to Stage III) during the hospital stay (*n* = 14) showed mean AIFELL scores at the day of admission to ICU of 5 ± 0.68.

The ANOVA and post-hoc calculations were performed to substantiate group differences more clearly and verified significant differences of evaluated score component values between different score values (Table 2). We found, for example, that the group of patients with an AIFELL score of 5 points had higher paraclinical component values than the group with 2 points (*p* < 0.001). Using the whole array of AIFELL components, it could be shown that the group with 6 points had significantly higher AIFELL component values than the group with 3 points (*p* < 0.0001).

**Table 2:** ANOVA of intergroup differences of the AIFELL score groups (1–6) using objective paraclinical measurements or the whole array of AIFELL components.

	**Paraclinical measurements**			**Whole array of AIFELL components**		
**AIFELL score by frontline experiences**			**ANOVA/Scheffe‘s post-hoc test**			**ANOVA/Scheffe‘s post hoc test**
Σ of positive components = points	**Mean ± SD**	**n**	**df = 116, F = 10.55, p < 0.0001**	**Mean ± SD**	**n**	**df = 116, F = 31.525, p < 0.0001**
**1**	−0.54 ± 0.28	12	1 < 2; 1 < 3; 1 < ***4; 1 < *5; 1 < **6	0.79 ± 0.93	12	1 < *4; 1 < *5; 1 < *6; 1 < 2; 1 < 3
**2**	−0.21 ± 0.43	26	2 < 3; 2 < 4; 2 < *5; 2 < 6	1.18 ± 0.55	26	2 < 3; 2 < *4; 2 < * 5; 2 < *6
**3**	−0.04 ± 0.32	16	3 < 5; 3 < 6	1.66 ± 0.67	16	3 < **5; 3 < *6;
**4**	0.01 ± 0.65	31	4 < **5; 4 < 6	2.21 ± 0.93	31	4 < 5; 4 < *6
**5**	0.52 ± 0.42	24		2.77 ± 0.83	24	
**6**	0.30 ± 0.38	8		4.30 ± 0.38	8	

*Legend*: Paraclinical measurements = *z*-standardized mean values of serum LDH, CRP, inverse absolute lymphocyte count and temperature measured auricularly. Whole array of AIFELL components = sum of paraclinical measurements, lung infiltrates and altered smell or taste. *p < .001; **p < .01; ***p = .04. In only 86 of the 122 included cases, LDH values were determined. Smell and taste alterations were actively mentioned by the patients and not routinely asked by the physicians. Therefore, the number of positive cases is only 19. Patients without any positive components relating to the AIFELL score (Σ0, *n* = 5) were not included in statistical group comparisons. LDH, lactate dehydrogenase; CRP, C-reactive protein; SD, standard deviation.

Based on the evaluation of the initial data of 30 patients, we generated the AIFELL score as a simple triage instrument for the ER setting consisting of frequently available elements like patient symptoms (fever, altered smell or taste), laboratory tests (differential blood count, CRP, LDH) and imaging. Afterwards, we evaluated its diagnostic performance in a larger number of consecutive patients hospitalized for suspected COVID-19.

A host risk score dealing with comorbidities of COVID-19 patients^7^ as well as scores predicting critical illness^8^ or hyperinflammation^9^ in hospitalized patients with proven COVID-19 have been previously published. However, no ER triage score to identify probable COVID-19 cases in more critical stages (II and III) has been proposed yet. The AIFELL score uses only frequently obtained data usually available both, in the ER and the general practice setting. Other additional laboratory parameters like ferritin, troponin^10^ and D-dimers,^11^ which are elevated in severe cases of COVID-19,^12^ as well as more sophisticated parameters such as interleukin (IL)-6^11^ and soluble IL-2 receptor^13^ may also be of interest, but are not routinely evaluated or are not widely available. Therefore, these additional parameters are less applicable for ER or general practice triage purposes.

During the COVID-19 pandemic and partly scarce medical resources, the AIFELL score may be useful for selecting symptomatic COVID-19 cases from patients with rather unspecific general or respiratory symptoms in the ER or general practice setting who should immediately get a SARS-CoV-2 swab due to higher probability of the disease. The score is not intended to identify asymptomatic or oligosymptomatic SARS-CoV-2 infections (COVID-19 Stage I), which generally can be dealt with in the outpatient setting.

The major limitation of this work is the single-center evaluation of only a limited number of patients. Due to its retrospective nature, some values were not obtained from all patients. For example, in 36 of all included cases LDH values were missing as it was not measured in every admitted patient. Smell and taste alterations were actively mentioned by the patients and not routinely asked by the physicians during the study period.

The strengths of the AIFELL score are its simplicity, immediate availability as well as wide applicability due to simple components. The AIFELL score obviously needs to be prospectively applied in larger cohorts of patients to gain more reliable data regarding its diagnostic yield.

## Author contributions

IL: idea, data collection, assembly and interpretation, statistical analysis, manuscript writing; EU: statistical expertise, advice and analysis, manuscript writing; EB: idea, manuscript correction and approval; MMS: idea, analysis and interpretation of data, manuscript writing, correction and approval.

IL and MMS take the responsibility for the paper as a whole.

## Conflicts of interest

The authors declare no conflicts of interest.

## References

[1] Sheridan C. (2020). Fast, portable tests come online to curb coronavirus pandemic. Nat Biotechnol.

[2] Siddiqi H.K., Mehra M.R. (2020). COVID-19 illness in native and immunosuppressed states: a clinical-therapeutic staging proposal. J Hear Lung Transplant.

[3] Zhou F., Yu T., Du R. (2020). Clinical course and risk factors for mortality of adult inpatients with COVID-19 in Wuhan, China: a retrospective cohort study. Lancet.

[4] Rodriguez-Morales A.J., Cardona-Ospina J.A., Gutiérrez-Ocampo E. (2020). Clinical, laboratory and imaging features of COVID-19: a systematic review and meta-analysis. Travel Med Infect Dis.

[5] Huang C., Wang Y., Li X. (2020). Clinical features of patients infected with 2019 novel coronavirus in Wuhan, China. Lancet.

[6] Lechien J.R., Chiesa-Estomba C.M., De Siati D.R. (2020). Olfactory and gustatory dysfunctions as a clinical presentation of mild-to-moderate forms of the coronavirus disease (COVID-19): a multicenter European study. Eur Arch Otorhinolaryngol.

[7] Shi Y., Yu X., Zhao H., Wang H., Zhao R., Sheng J. (2020). Host susceptibility to severe COVID-19 and establishment of a host risk score: findings of 487 cases outside Wuhan. Crit Care.

[8] Liang W., Liang H., Ou L. (2020). Development and validation of a clinical risk score to predict the occurrence of critical illness in hospitalized patients with COVID-19. JAMA Intern Med.

[9] Mehta P., Mcauley D.F., Brown M. (2020). Correspondence COVID-19: consider cytokine storm syndromes and. Lancet.

[10] Lippi G., Lavie C.J., Sanchis-Gomar F. (2019). Cardiac troponin I in patients with coronavirus disease 2019 (COVID-19): evidence from a meta-analysis. Prog Cardiovasc Dis 2020.

[11] Gao Y., Li T., Han M. (2020). Diagnostic utility of clinical laboratory data determinations for patients with the severe COVID-19. J Med Virol.

[12] Velavan T.P., Meyer C.G. (2020). Mild versus severe COVID-19: laboratory markers. Int J Infect Dis.

[13] Chen G., Wu D., Guo W. (2020). Clinical and immunologic features in severe and moderate forms of Coronavirus Disease 2019. J Clin Invest 2020.

