# Supplementary material for: Triage tool for suspected COVID-19 patients in the emergency room: AIFELL score
Source: Braz J Infect Dis. 2020 Aug 20;24(5):458–61. doi: 10.1016/j.bjid.2020.07.003 (PMC7440000; doi:10.1016/j.bjid.2020.07.003)
Supplement: Supplementary file 1 [file mmc1.doc]

**AIFELL Score: Clinical Protocol**

Criteria met at initial presentation in the emergency room (ER). The AIFELL score can be calculated for any patient presenting with respiratory or general symptoms related to COVID-19 (cough, dyspnea, sore throat, fever, fatigue, myalgias, less common are rhinorrhea, sputum production, diarrhea, abdominal pain).

| **Components of the AIFELL score** | **Points** |
| --- | --- |
| **Altered sense of smell/taste**  Any changes or impairments of the sense of smell/taste since onset of any COVID-19-related symptoms or directly before onset of respiratory/general symptoms. | **Yes** / No (**1** / 0 points) |
| **Inflammation (C-reactive protein ≥ 30 mg/l)** | **If CRP ≥ 30 mg/l = 1 point**, else 0 points. |
| **Radiological Infiltrates**  Any radiological infiltrates in chest X-ray or thoracic CT (no differentiation between unilateral or bilateral infiltrates). | **Yes** / No (**1** / 0 points) |
| **F**ever (≥ 38.0°C)  Auricular measurement or other accepted methods (anal, axillary). In our setting, auricular measurements were performed and the cut-off was determined to be ≥ 38.0°C. Other measurement locations are acceptable, but may have different cut-off values and should be a standardized in all patients (i. e. only axillar or anal measurements should be performed). The patients should be asked whether he/she took any antipyretics before coming to the ER. If antipyretics were taken, the patients should be asked regarding his/her body temperature before taking antipyretics (elevated?) and the patient should be checked for an increase of body temperature ≥ 38.0°C during the first 24h of hospitalization. Counting a body temperature < 38.0°C at presentation in the ER after the patient took antipyretics may lead to false-negative results. | **Yes**, if temperature at presentation is ≥ 38.0°C OR temperature before taking antipyretics was ≥ 38.0°C OR temperature increase during the first 24h of hospitalization ≥ 38.0°C = **1 points**,  else 0 points. |
| **E**levated **L**actate dehydrogenase (LDH) levels (> 400 U/l)  Test performed using Roche "Cobas 8000" platform in our setting  Eur. J. Clin. Chem. Clin. Biochem. 1986;24:409-432, Clin. Chem. Lab. Med. 2002;40(6):643-648  Method: UV test at 37°C, usual range (240 - 480 U/l) | **Yes** / No (**1** / 0 points) |
| Lymphocytopenia (absolute count < 1.45 G/l)  Only the absolute count (not the relative count) is relevant. | **Yes** / No (**1** / 0 points) |

The score is calculated by counting the number of criteria met at initial presentation in the ER, whereas each criterion equals one point (Score range 0 to 6 points).

A score of ≥ 4 points/criteria met at presentation was highly associated with qPCR-based SARS-CoV-2 detection in nasopharyngeal swabs and presence of symptomatic COVID-19 (Stages II or III^1^), thus justifying hospitalization. Scores between 0 and 3 were associated with other respiratory conditions

The score is not intended for asymptomatic or oligosymptomatic patients (COVID-19 Stage I^1^), who may also be SARS-CoV-2 positive.

| **AIFELL score**  **(points)** | **Interpretation** |
| --- | --- |
| 4 – 6 | High probability of COVID-19 (Stages II and III), **SARS-CoV-2 swab is necessary.** |
| 0 – 3 | Lower probability of COVID-19 (Stages II and III)  Respiratory symptoms likely due to other medical condition. |

**References**

1. Siddiqi HK, Mehra MR. COVID-19 Illness in Native and Immunosuppressed States: A Clinical-Therapeutic Staging Proposal. *J Hear Lung Transplant*. March 2020. doi:10.1016/j.healun.2020.03.012

**AIFELL Score: Selection of score variables from the pilot population**

The parameters were established based on observations of a pilot cohort of 30 consecutively encountered patients with qPCR-proven COVID-19, who were hospitalized in the Department of Internal Medicine at the University Hospital Zurich through the emergency room (ER) in March 2020 and whose laboratory parameters showed characteristic patterns:

- 24 patients (80%) had elevated C-reactive protein levels (CRP), which is measured as a typical marker of inflammation (median value 77.5, range 2.0 – 380 in mg/l with a normal range < 5 mg/l). The lowest value for patients classified as having COVID-19 Stage 2 was 30 mg/l. Thus, this value was chosen as the cut-off.
- 28 patients (93%) had lymphocytopenia in the differential blood count (median 0.9, range 0.2 – 19.7 in G/l, absolute count, normal range 1.5 – 4.0 G/l). The highest value of 19.7 G/l in the pilot population was due to the inclusion of a patient with chronic lymphocytic leukemia. Comorbidities were not an exclusion criterion in patients with a positive SARS-CoV-2 qPCR swab result. Besides this outlier, the highest value in patients presented with lymphocytopenia in the initial pilot population was slightly below 1.45 G/l, which was chosen as the cut-off value.
- 18 patients (60%) had an elevated lactate dehydrogenase (LDH, median 491.0, range 295.0 – 999.0 in U/l, normal range 240 – 480 U/l). The lowest value for patients classified as having COVID-19 Stage 2 was 387 U/l. However, most patients with COVID-19 Stage 2 in the pilot study had values > 400 U/I, which was chosen as the cut-off value.

CRP measurements and a differential blood count are routinely determined in every patient presenting with signs of inflammation in the ER of the University Hospital Zurich.

We observed that 26 patients in the pilot population (87%) had unilateral or bilateral pulmonary infiltrates in conventional radiography as confirmed by radiologists. Coincidentally, 26 patients (87%) presented with fever (median 38,6, range 36.4 – 39.7). However, not all patients with pulmonary infiltrates had fever.

The patients in the pilot population could be initially classified to either COVID-19 Stage I (N = 4) or Stage II (N = 26) at the moment of hospitalization.

When the question of an ER triage score arose, literature searches for typical COVID-19-associated laboratory parameters as described were performed and early published studies (references 3 – 5 in the manuscript)^1–3^ confirmed our observational findings as characteristics of COVID-19.

Based on the described observations and literature searches, the mentioned laboratory parameters, pulmonary infiltrates as a sign of inflammation and fever were included in the emerging score. 5 of 30 patients mentioned actively an attenuation of taste or smell. As this COVID-19-associated symptom was not yet widely recognized, but already discussed in the literature^4^, it was not routinely asked by attending physicians but included into the emerging triage score as more evidence about smell and taste alterations in COVID-19 were published. To make the triage score easy to use and to calculate by attending ER physicians during the COVID-19 pandemic, each component was weighted equally with 1 point (thus the score range of 0 to 6 points) and the acronym ‘AIFELL’ was coined.
